# A Case of Hypercalcemic-Type Stage IVB Small-Cell Carcinoma of the Ovary in a Young Woman

**DOI:** 10.7759/cureus.59214

**Published:** 2024-04-28

**Authors:** Omeed Partovi, Ramsey Rayes, Britta L Bureau, Stephanie Strohbeen, Nisar Asmi

**Affiliations:** 1 Department of Internal Medicine, Medical College of Wisconsin, Milwaukee, USA; 2 Department of Neurology, Medical College of Wisconsin, Milwaukee, USA; 3 Section of Hospital Medicine, Division of General Internal Medicine, Medical College of Wisconsin, Milwaukee, USA

**Keywords:** small cell carcinoma of the ovary, ovary cancer, rare cancers, young woman, malignancy related hypercalcemia

## Abstract

A 38-year-old nulliparous woman with severe obesity (BMI 66) and hypertension presented with constipation, fatigue, weakness, and poor appetite that had progressively worsened over the prior two to three weeks. Upon admission, the patient was found to have significant hypercalcemia, leukocytosis, and lactic acidosis. Computed tomography (CT) scan of the chest, abdomen, and pelvis revealed an adnexal mass with extensive lesions throughout her pelvis, abdomen, and chest. An ultrasound-guided omental core biopsy was performed, which was confirmatory for metastatic ovarian small cell carcinoma. Given her poor prognosis and clinical status, chemotherapy was likely to provide minimal benefit and ultimately the patient decided to pursue a comfort-oriented plan of care and passed away on day 9 of admission.

## Introduction

Small cell carcinoma of the ovary, hypercalcemic type (SCCOHT) is a very rare (<0.01% of ovarian malignancies) and highly malignant ovarian cancer that predominantly affects young women [1]. The mean age of diagnosis is 23 years and the long-term survival rate in early-stage cases is around 30%, with the majority of patients dying within two years of diagnosis [1,2]. There are fewer than 500 reported cases of SCCOHT in the current literature and an established treatment regimen for the malignancy does not currently exist [1,2].

## Case presentation

**Figure 1:**
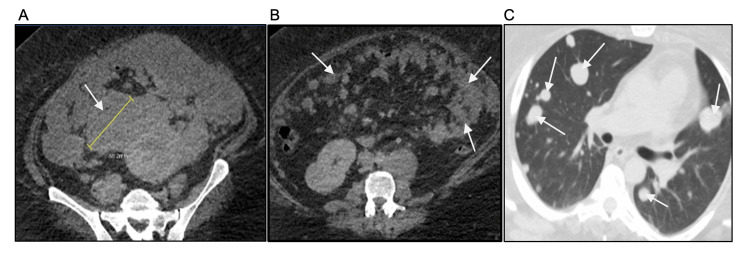
CT chest, abdomen, and pelvis (CT CAP) revealing an adnexal mass with extensive lesions A: 8.8 cm adnexal mass of soft tissue density in right hemipelvis (CT A/P). B: Extensive peritoneal metastases and omental caking (CT A/P). C: Innumerable bilateral metastatic lung nodules (CT chest). A/P: abdomen and pelvis.

**Figure 2:**
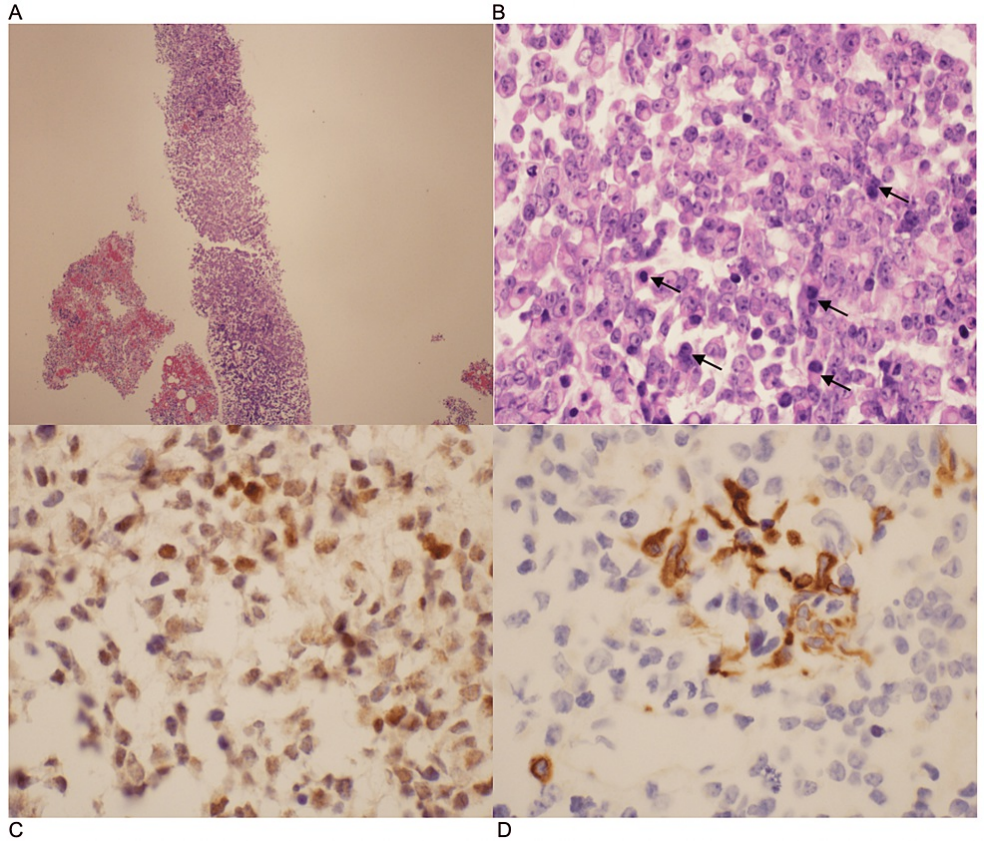
Pathology slide images of omental core biopsy A: Needle core biopsy specimen, completely replaced by high-grade malignant neoplasm (H&E @ 100x mag). B: Neoplastic cells are monotonous, with a moderate amount of cytoplasm, uniform round nuclei, and prominent nucleoli (H&E @ 400x mag). C: Tumor cells express PAX 8 nuclear reactivity. D: Tumor cells with focal wide-spectrum cytokeratin reactivity.

A 38-year-old nulliparous woman with morbid obesity (BMI 66) and hypertension presented with constipation, fatigue, weakness, and poor appetite that had progressively worsened over the past 2-3 weeks. She came to the emergency department after falling onto her bed from lightheadedness without loss of consciousness. She denied having any chest pain and had no family history of cancer or any history of substance use. Upon admission, the patient was found to have significant hypercalcemia (17.1 mg/dL, reference range [rr] 8.5-10.5), leukocytosis (29.4 x 10^3^/mm^3^, rr 4.5-11.0), and lactic acidosis (5.4 mmol/L, rr 0.5-2.2). On examination, she appeared fatigued, had increased heart rate and work of breathing, diffuse tenderness to her lower quadrants bilaterally, a hardened pannus in the lower abdomen, decreased capillary refill, 1+ lower extremity edema, facial hair noted on her chin, and was alert and oriented to person, place, and time. Her initial workup for hypercalcemia showed low parathyroid hormone (PTH) (8.4 pg/mL, rr 10-60), 25-hydroxycholecalciferol (12.7 ng/mL, rr 20-40), and 1,25-dihydroxycholecalciferol (<5.0 pg/mL, rr 18-78) and elevated parathyroid hormone-related protein (PTHrP) (25 pmol/L, rr < 2.5). CA-125 (262 kU/L, rr 0-35) and lactate dehydrogenase (621 U/L, rr 125-214) were also elevated. All her labs for kidney function were normal. While working up the etiology of the patient’s symptoms, the patient’s hypercalcemia improved after receiving IV fluids, calcitonin, and zoledronic acid. Computed tomography (CT) scan of the chest, abdomen, and pelvis (CAP) revealed an 8.8 cm adnexal mass with extensive lesions throughout her pelvis, abdomen, and chest (Figure 1). Of note, a CT CAP from 10 months prior did not demonstrate any signs of malignancy. An ultrasound-guided omental core biopsy was performed which was confirmatory for metastatic ovarian small cell carcinoma (immunohistochemistry positive for PAX 8, focally positive for CK, EMA, WT1, and calretinin, and negative for CK7, CK20, TTF-1, CDX2, CD10, synaptophysin, chromogranin, and inhibin) (Figure 2).

Given her high-risk profile, poor prognosis, clinical status, and poor performance status, chemotherapy was unlikely to benefit her. Ultimately, the patient decided to pursue a comfort-focused plan of care and ultimately died on day 9 of admission.

## Discussion

While this is the first case to report clinically staged IVB small-cell carcinoma of the ovary, hypercalcemic type (SCCOHT), some aspects of this malignancy, such as tumor cells expressing PTHrP, have been described in the literature since 1979 [3,4]. However, treatment strategies for SCCOHT did not start developing until 2014, when recurrent deleterious mutations were discovered in the SMARCA4 gene in greater than 95% of SCCOHT cases. Since this discovery, collaborative research efforts to develop a consensus guideline for the treatment in the management of SCOOHT have been ongoing internationally [2,5].

The International Federation of Gynecology and Obstetrics (FIGO) guidelines for ovarian cancer are currently used to stage SCCOHT. Cases with FIGO stage 1A currently have the best prognosis with a five-year overall survival rate of 33% [4,6,7]. Based on small case reports and case series, additional risk factors that correlate with a better prognosis include age greater than 30 years, calcium level within normal limits, and tumor size less than 10 cm.

Current cases in the literature that describe patients with earlier stages of the malignancy typically undergo a multimodal therapy approach involving cytoreductive surgery, radiotherapy, and platinum-based chemotherapy. The medication regimen is largely determined from data related to the outcomes of patients with small-cell lung carcinoma that had good outcomes [6,8]. Despite these multimodal treatment regimens in patients with an early diagnosis of SCCOHT, the long-term survival rate is still only around 30%. To improve these outcomes, research is largely focused on uncovering target immunotherapy interventions that can regulate SMARCA4 expression as a first-line adjuvant to chemotherapy [2].

Because our patient presented with stage IV disease, surgery was not a feasible treatment option [2]. While a cisplatin-based chemotherapy regimen could have been a potential option, due to the extensive burden of disease and poor functional status, the patient decided to pursue comfort-oriented care. A rapid decline in respiratory status led her to pass away nine days later. Additionally, our patient’s CT scan from 10 months prior to admission that was without any evidence of malignancy demonstrates the extremely aggressive nature of this disease. By reporting this case, we hope to increase awareness of this rare malignancy, highlight its aggressive nature, and emphasize the need for early diagnosis.

## Conclusions

We present a case of clinically staged type IVB SCCOHT to emphasize the importance of considering this rare malignancy in young women who present with unexplained hypercalcemia. Our case is unique in that our patient presented with an advanced stage of SCCOHT that has not been reported before in the literature to our knowledge. By sharing our patient’s clinical findings and the extremely aggressive course of their malignancy, we hope to increase awareness of the malignancy for earlier detection and better characterization of its progression. We hope this shall encourage further research efforts toward more effective treatment strategies that improve clinical outcomes and survival.
